# Transcutaneous Carbon Dioxide Monitoring More Accurately Detects Hypercapnia than End-Tidal Carbon Dioxide Monitoring during Non-Intubated Video-Assisted Thoracic Surgery: A Retrospective Cohort Study

**DOI:** 10.3390/jcm12041706

**Published:** 2023-02-20

**Authors:** Hyun Jung Lee, Jae Hee Woo, Sooyoung Cho, Sunyoung Moon, Sook Whan Sung

**Affiliations:** 1 Department of Anesthesiology and Pain Medicine, College of Medicine, Ewha Womans University, 260, Gonghang-daero, Gangseo-gu, Seoul 07804, Republic of Korea; 2 Department of Anesthesiology and Pain Medicine, Ewha Womans University Mokdong Hosptial, 1071, Anyangcheon-ro, Yangcheon-gu, Seoul 07985, Republic of Korea; 3 Department of Thoracic and Cardiovascular Surgery, Ewha Womans University Seoul Hospital, 260, Gonghang-daero, Gangseo-gu, Seoul 07804, Republic of Korea

**Keywords:** transcutaneous carbon dioxide monitoring, end-tidal carbon dioxide monitoring, hypercapnia, non-intubated video-assisted thoracoscopic surgery

## Abstract

Transcutaneous carbon dioxide (PtcCO_2_) monitoring is known to be effective at estimating the arterial partial pressure of carbon dioxide (PaCO_2_) in patients with sedation-induced respiratory depression. We aimed to investigate the accuracy of PtcCO_2_ monitoring to measure PaCO_2_ and its sensitivity to detect hypercapnia (PaCO_2_ > 60 mmHg) compared to nasal end-tidal carbon dioxide (PetCO_2_) monitoring during non-intubated video-assisted thoracoscopic surgery (VATS). This retrospective study included patients undergoing non-intubated VATS from December 2019 to May 2021. Datasets of PetCO_2_, PtcCO_2_, and PaCO_2_ measured simultaneously were extracted from patient records. Overall, 111 datasets of CO_2_ monitoring during one-lung ventilation (OLV) were collected from 43 patients. PtcCO_2_ had higher sensitivity and predictive power for hypercapnia during OLV than PetCO_2_ (84.6% vs. 15.4%, *p* < 0.001; area under the receiver operating characteristic curve; 0.912 vs. 0.776, *p* = 0.002). Moreover, PtcCO_2_ was more in agreement with PaCO_2_ than PetCO_2_, indicated by a lower bias (bias ± standard deviation; −1.6 ± 6.5 mmHg vs. 14.3 ± 8.4 mmHg, *p* < 0.001) and narrower limit of agreement (−14.3–11.2 mmHg vs. −2.2–30.7 mmHg). These results suggest that concurrent PtcCO_2_ monitoring allows anesthesiologists to provide safer respiratory management for patients undergoing non-intubated VATS.

## 1. Introduction

In a modern era of minimally invasive procedures, non-intubated video-assisted thoracoscopic surgery (VATS) has been introduced in an effort to reduce the adverse effects of general anesthesia and tracheal intubation in conventional VATS. Non-intubated VATS has been demonstrated in several thoracic surgeries, from minor procedures such as pleural, lung, or mediastinal biopsies, resections of peripheral nodules, and thymectomies to major pulmonary resections [1]. The outcomes of these operations have encouraged further use of non-intubated anesthetic techniques in selected patients [2,3,4].

Intravenous anesthesia combined with a regional block—such as the intercostal nerve or paravertebral block—is a commonly used anesthetic protocol for non-intubated VATS [5]. Respiratory management in patients who undergo non-intubated one-lung ventilation (OLV) with sedation is challenging for anesthesiologists because ventilation cannot be mechanically controlled in these patients. Although oxygenation is typically well maintained, maintaining an appropriate partial pressure of arterial carbon dioxide (PaCO_2_) during non-intubated VATS is a demanding task. A “permissive hypercapnia” strategy (PaCO_2_ up to 70 mmHg), regarded as generally tolerable, is typically implemented [5]. This means that immediate adjustment of the infusion rates of anesthetic drugs in conjunction with manual ventilation is required when PaCO_2_ rises to >60 mmHg to maintain it within the permissive range [6]. If PaCO_2_ exceeds 80 mmHg, despite efforts to reverse hypercapnia, conversion to general anesthesia with endotracheal intubation should be considered [5]. Detecting an increase in PaCO_2_ to >60 mmHg is thus crucial to preventing excessive hypercapnia.

While the gold standard for determining hypercapnia during general anesthesia is the measurement of PaCO_2_ by arterial blood gas analysis (ABGA), this method is invasive and intermittent. End-tidal CO_2_ partial pressure (PetCO_2_) monitoring is preferred for the noninvasive and continuous monitoring of PaCO_2_. When patients breathe spontaneously during non-intubated VATS, a nasal PetCO_2_ monitoring device connected to the nasal cannula is commonly used. However, it has an inherently limited ability to accurately measure PaCO_2_ compared to devices connected to the endotracheal tube due to increased dead space, which could be exacerbated during OLV [7]. Due to this limitation of nasal PetCO_2_ monitoring, transcutaneous CO_2_ partial pressure (PtcCO_2_), which continuously measures PaCO_2_ through arterialized capillary blood in tissues, has been suggested as a reliable tool to detect hypercapnia during invasive procedures requiring moderate-to-deep sedation that can induce hypoventilation [8,9,10]. In addition, the previous studies showed that PtcCO_2_ could be superior to PetCO_2_ monitoring during OLV, which induces a ventilation-perfusion mismatch that lowers the accuracy of PetCO_2_ monitoring [1,11].

To date, PtcCO_2_ monitoring during non-intubated VATS has not been sufficiently validated. Notably, the intraoperative hypotension, especially by a sudden deepening of anesthesia after intercostal and vagal block, lateral decubitus positioning, and mediastinal shift (caused by iatrogenic pneumothorax and the CO_2_ gas inflation to facilitate lung collapse and improve the visual field) are conditions frequently confronted in non-intubated VATS and can influence the accuracy of PtcCO_2_ monitoring [12]. In addition, vasoconstrictors administered to treat intraoperative hypotension that may lead to peripheral hypoperfusion, and consistently higher PaCO_2_ levels during non-intubated VATS, could reduce the accuracy of PtcCO_2_ monitoring, altogether necessitating its validation in non-intubated VATS before routine application [11,13]. Therefore, we aimed to compare the accuracy of two noninvasive (PtcCO_2_ and PetCO_2_) monitoring methods and evaluate the predictive power and sensitivity when using PaCO_2_ > 60 mmHg as the threshold at which respiratory intervention should be initiated to prevent the development of excessive hypercapnia.

## 2. Materials and Methods

### 2.1. Study Design and Participants

We retrospectively reviewed electronic medical records of patients who underwent non-intubated VATS to treat lung cancer between December 2019 and May 2021 at Ewha Womans University Seoul Hospital (Seoul, Republic of Korea). While we did not have specific exclusion criteria for this retrospective analysis, our institutional acceptance criteria for non-intubated VATS excluded patients with the following conditions at the planning stage of their surgery: (i) body mass index (BMI) > 30 kg/m^2^; (ii) requirement for vasopressors to maintain a mean arterial blood pressure greater than 65 mmHg; (iii) anticipated difficult airway management, neuromuscular disease, phrenic nerve palsy, persistent cough, or persistent sputum; (iv) anticipated severe adhesion of the operated lung, (v) a history of thoracic surgery; and (vi) any contraindication for permissive hypercapnia, such as increased intracranial pressure and right ventricular failure.

### 2.2. Data Acquisition

All data were extracted from electronic medical records, including anesthesia records. Data on the following demographic characteristics were extracted: age, sex, height, body weight, American Society of Anesthesiologists (ASA) physical status, and results of pulmonary function test. For anesthetic and procedure associated data, the following were extracted: the duration of anesthesia, administration of anesthetics and vasoconstrictor agents, and type of thoracic procedure. For CO_2_ data, we collected datasets of intraoperative PetCO_2_, PtcCO_2_, and PaCO_2_ that were simultaneously measured. Our institutional protocol was set to measure PaCO_2_ at time points as follows: during two-lung ventilation (TLV) preoperatively, 15 min after OLV by creating iatrogenic pneumothorax, after lobectomy, and upon PetCO_2_ > 55 mmHg or PtcCO_2_ > 60 mmHg. For cases of conversion to general anesthesia with tracheal intubation performed during surgery, the CO_2_ datasets acquired prior to the conversion were included in the analysis.

### 2.3. The Protocol of Anesthesia for Non-Intubated VATS

The below protocol was followed for anesthesia for non-intubated VATS performed at our institution.

#### 2.3.1. Induction and Maintenance of Anesthesia for Non-Intubated VATS

Standard ASA monitoring was applied during the surgery. On arrival at the operating theater, patients were administered 5 mg of dexamethasone and 0.2 mg of glycopyrrolate. Spontaneous breathing was maintained throughout the non-intubated VATS procedure. Continuous dexmedetomidine infusion was administered to all patients at a rate of 0.5–0.7 μg/kg/h following 10 min of a loading dose of 1 μg/kg. Propofol administration was initiated with effective site concentrations of 3.0 μg/mL and titrated to 2.0–4.0 μg/mL. In initially awake patients, the dose was titrated to achieve a modified Ramsay sedation (MRS) score between 4 (appears asleep; purposeful responses to verbal commands louder than a usual conversation or to light glabellar tap) and 5 (asleep; sluggish purposeful responses only to loud verbal commands or strong glabellar tap). After an appropriate sedation level was achieved based on MRS score, the bispectral index was monitored using electroencephalographic analysis (target at levels between 40 and 60) to ensure an adequate sedation level during the surgery. Remifentanil was simultaneously initiated at 0.5 ng/mL and titrated to within a range of 0.5–3.0 ng/mL to maintain a respiratory rate of ≥10 breaths/min.

#### 2.3.2. CO_2_ Measurements during Non-Intubated VATS

**Figure 1:**
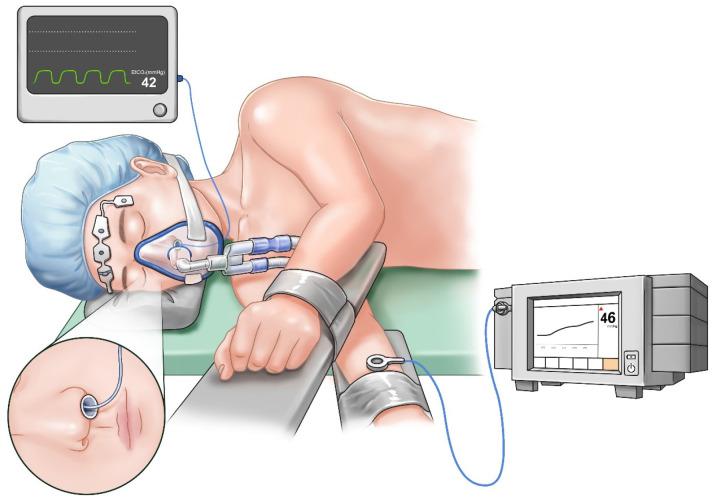
Schematic illustration of nasal end-tidal carbon dioxide (PetCO_2_) and transcutaneous carbon dioxide (PtcCO_2_) monitoring. An end-tidal sample line was inserted into the nasopharyngeal airway to monitor PetCO_2_. A transcutaneous monitoring probe was placed on the forearm ipsilateral to the non-operated lung to monitor PtcCO_2_.

Once a satisfactory sedation level was achieved, a nasopharyngeal airway was inserted. PetCO_2_ monitoring was performed using an infrared CO_2_ analyzer (Avance CS^2^, GE Healthcare, Madison, WI, USA) by inserting a sample line into the nasopharyngeal airway to minimize the potential under-detection of exhaled gas due to airway obstruction (Figure 1). The radial artery on the non-operated side was cannulated to monitor continuous arterial blood pressure and sample arterial blood for gas analysis. PtcCO_2_ was measured using a TCM4^TM^ device (Radiometer, Copenhagen, Denmark). The transcutaneous monitoring technique was standardized by applying a probe on the forearm ipsilateral to the non-operated lung in the lateral decubitus position (Figure 1). Before placement, the device was calibrated ex vivo as per the manufacturer’s recommendations. Then, the skin surface where the electrode was placed was swabbed with alcohol to facilitate disc adhesion. Subsequently, the probe was mounted on the electrode with the working temperature set to 42 °C to arterialize the capillary blood flow in the skin. The subsequent in vivo calibration was based on the results of the first ABGA performed after a 10-min equilibration period from the time of the placement of probe on the patient for stabilization of the measurement [14,15].

#### 2.3.3. Respiratory Management during Non-Intubated VATS

Six liters per minute of oxygen were supplied via a facial mask to maintain percutaneous oxygen saturation (SpO_2_) at ≥90%. The facial mask was secured to the patients with an elastic strap, but a full fit was not ensured to allow the collapse of the non-dependent lung. When desaturation (SpO_2_ < 90%) occurred, the patient was first assessed for airway obstructions, which were relieved by chin lifting and head tilting maneuvers. If desaturation persisted, the sedation level was titrated by adjusting the doses of the sedatives, and manual Ambu-bagging was applied as needed. A decision for conversion to general anesthesia was made in the following situations: an uncontrolled vigorous diaphragmatic movement that hampered the surgical procedure, persistent hypoxemia (SpO_2_ < 90%) and/or excessive hypercapnia (PaCO_2_ > 80 mmHg) despite the respiratory management described above, persistent cough, unstable hemodynamics, and conversion to open thoracotomy. Atropine was administered when the heart rate was < 50 beats/min. In addition, an ephedrine bolus or norepinephrine infusion was administered when the systolic blood pressure was <90 mmHg.

### 2.4. Techniques for Non-Intubated VATS

In all surgeries, uniportal thoracoscopic lobectomy and mediastinal node dissection were performed by a team of surgeons who used the same standardized technique for non-intubated VATS. The patients were placed in the lateral decubitus position. First, a 1:1 mixture of 0.75% ropivacaine and 2% lidocaine was infiltrated into the skin and subcutaneous tissue, followed by a 4 cm incision made along the anterior axillary line of the fourth or fifth intercostal space. Subsequently, iatrogenic pneumothorax was generated by creating an incision through the chest wall and pleura, which caused the ipsilateral lung to collapse gradually (note that surgeons at our institution do not use the CO_2_ gas inflation technique to facilitate lung collapse). The surgeon then performed an intercostal nerve block from the third to the sixth intercostal space. Additionally, a vagal block on the corresponding side was made with 2 mL of a 1:1 mixture of 0.75% ropivacaine and 2% lidocaine for each nerve under direct visualization through a thoracoscope. On completion of the surgical procedure, the intercostal nerve block was repeated before the closure of the pleura for postoperative analgesia.

### 2.5. Statistical Analyses

Statistical analyses were performed using SPSS version 26.0 (IBM Corp., Armonk, NY, USA). The primary outcomes were the sensitivity and predictive power of two noninvasive CO_2_ monitoring methods (PetCO_2_ and PtcCO_2_) for hypercapnia (PaCO_2_ > 60 mmHg), which was the threshold level at which respiratory intervention was considered to correct hypercapnia. Values were dichotomized into PtcCO_2_ > 60 mmHg and PetCO_2_ > 55 mmHg. The criterion of PetCO_2_ > 55 mmHg was established in consideration of physiological alveolar dead space and the previous study [15]. They were then compared using the Yates corrected chi-squared method. Predictive power for PaCO_2_ > 60 mmHg was compared by constructing a receiver operating characteristic (ROC) curve and calculating the area under the curve (AUC). The secondary outcomes were the agreement between each measure (PetCO_2_ and PtcCO_2_) and PaCO_2_. The bias, i.e., the mean difference, between PaCO_2_ and noninvasive monitoring (PtcCO_2_ and PetCO_2_), precision (standard deviation [SD] of bias), and limit of agreement (LOA; bias ± 1.96SD) were calculated according to the Bland–Altman method. In addition, the relationship between the two noninvasive monitoring and PaCO_2_ were evaluated using linear regression analysis with the Pearson correlation coefficient (*r*).

In accordance with our retrospective study design, a post-power analysis, i.e., a two-ROC-curve power calculation, was conducted (with the pROC package of R, version 4.2.2) to verify whether our sample size was adequate to compare the predictive power of the two noninvasive monitoring methods. Measurements of PtcCO_2_ and PetCO_2_ and outcome values of 1 when hypercapnia (PaCO_2_ > 60 mmHg) occurred were entered into this calculation. The analysis yielded a power of 0.97, indicating that our sample size was adequate to evaluate the detection power of the two CO_2_ monitoring systems. Statistical significance was set to *p* < 0.05.

## 3. Results

### 3.1. Participant Characteristics

Fifty-four patients who underwent non-intubated VATS between December 2019 and May 2021 were assessed for eligibility; data of 11 patients (either PetCO_2_ or PtcCO_2_) were missing, and 43 patients were thus included in the analyses. The demographic characteristics of all participants are presented in Table 1. In addition, 2 of the 43 patients were converted to general anesthesia during surgery due to vigorous diaphragmatic movement; datasets of CO_2_ measurements before conversion were included for these patients. Consequently, 43 datasets of PaCO_2_, PetCO_2_, and PtcCO_2_ measurements were obtained preoperatively during TLV, and 111 datasets were obtained during OLV.

### 3.2. Primary Outcomes

**Figure 2:**
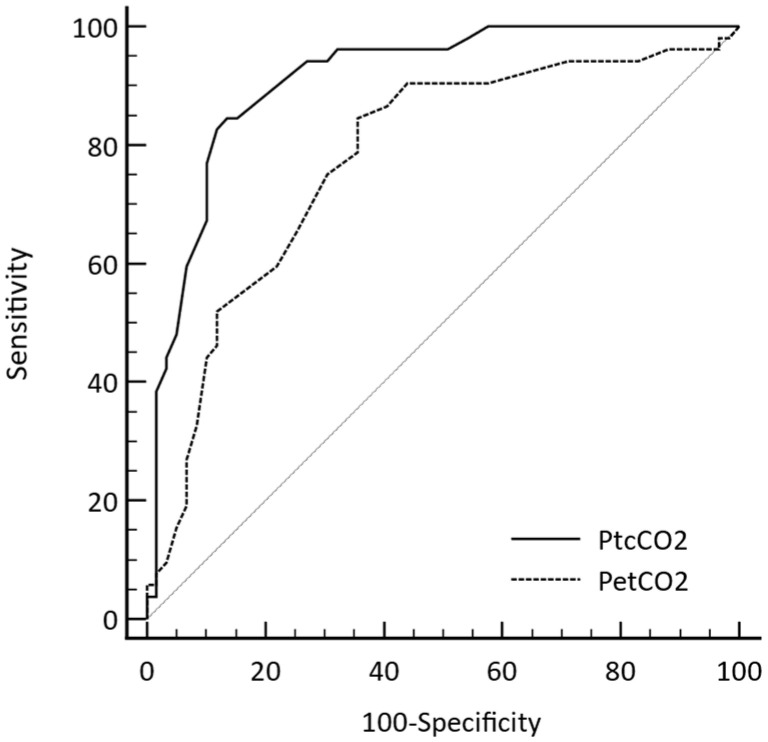
The receiver operating characteristic (ROC) curves comparing the predictive power of PtcCO_2_ and PetCO_2_ monitoring for hypercapnia (PaCO_2_ > 60 mmHg). PetCO_2_, end-tidal carbon dioxide partial pressure; PtcCO_2_, transcutaneous carbon dioxide partial pressure; PaCO_2_, partial pressure of arterial carbon dioxide.

During OLV, a PaCO_2_ > 60 mmHg was observed in 52 of 111 datasets. Using the predefined cutoff values of PtcCO_2_ > 60 mmHg and PetCO_2_ > 55 mmHg, the sensitivity of PtcCO_2_ monitoring to detect PaCO_2_ > 60 mmHg was significantly higher than that of PetCO_2_ (84.6% vs. 15.4%, *p* < 0.001). The predictive power of PtcCO_2_ for PaCO_2_ > 60 mmHg was higher than that of PetCO_2_ during OLV (AUC (95% confidence interval); 0.912 (0.843–0.957) vs. 0.776 (0.687–0.849), *p* = 0.002, Figure 2).

### 3.3. Secondary Outcome

**Figure 3:**
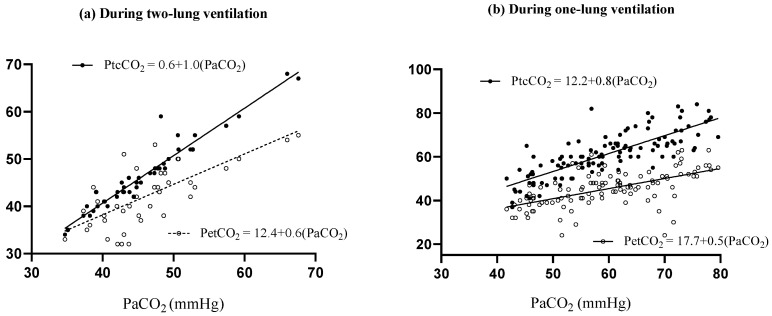
Correlation of PtcCO_2_ and PetCO_2_ with PaCO_2_ during (**a**) two-lung ventilation and (**b**) one-lung ventilation. PetCO_2_, end-tidal carbon dioxide partial pressure; PtcCO_2_, transcutaneous carbon dioxide partial pressure; PaCO_2_, partial pressure of arterial carbon dioxide.

The mean values of all physiological measures, including pH, PaO_2_, SpO_2_, PaCO_2_, PetCO_2_, and PtcCO_2_, during TLV and OLV, are listed in Table 2. The relationships of PtcCO_2_ and PetCO_2_ with PaCO_2_ during TLV and OLV are visualized in Figure 3a,b, respectively, and the Pearson correlation coefficients (*r*) were interpreted based on previous literature [16]. PtcCO_2_ measurements were very strongly correlated with PaCO_2_ as defined by the relationship *y* = 1.0*x* + 0.6 (*r* = 0.963, *p* < 0.001, Figure 3a). PetCO_2_ also showed a moderate correlation with PaCO_2_, described by the relationship *y* = 0.6*x* + 12.4 (*r* = 0.722, *p* < 0.001, Figure 3a). During OLV, PtcCO_2_ measurements were very strongly correlated with PaCO_2_: *y* = 0.8*x* + 12.2 (*r* = 0.801*, p* < 0.001, Figure 3b), and PetCO_2_ showed a fair correlation with PaCO_2_, indicated by the relationship *y* = 0.5*x* + 17.7 (*r* = 0.595, *p* < 0.001, Figure 3b).

**Figure 4:**
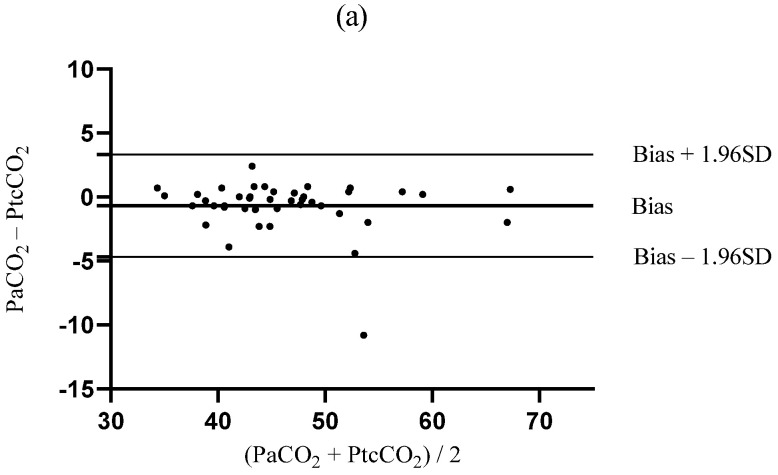

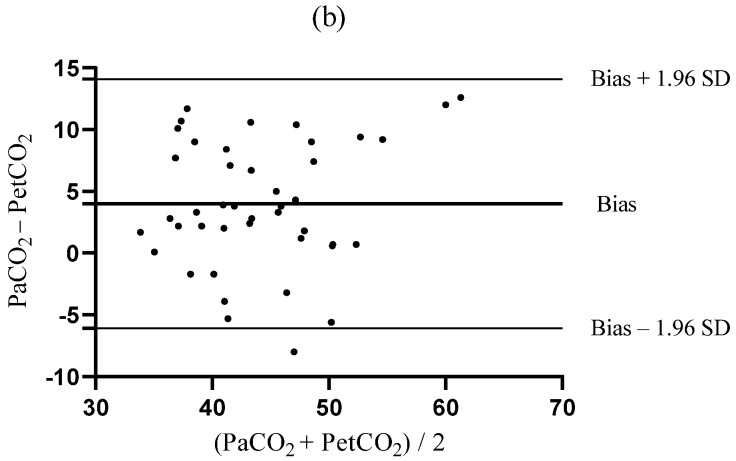
Bland–Altman plots comparing the agreement between (**a**) PaCO_2_ and PtcCO_2_ and (**b**) PaCO_2_ and PetCO_2_ during two-lung ventilation. PetCO_2_, end-tidal carbon dioxide partial pressure; PtcCO_2_, transcutaneous carbon dioxide partial pressure; PaCO_2_, partial pressure of arterial carbon dioxide; SD, standard deviation.

**Figure 5:**
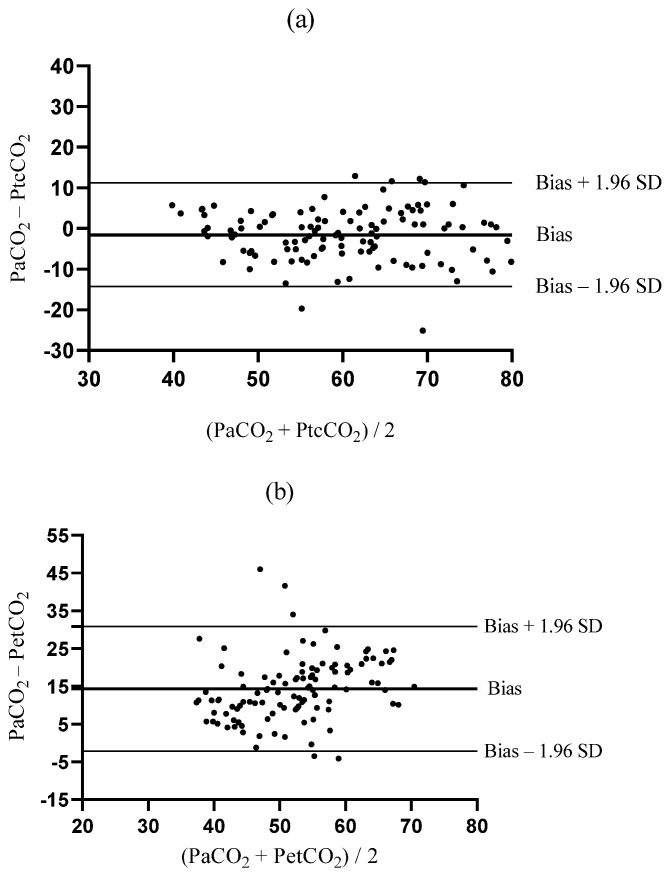
Bland–Altman plots comparing the agreement between (**a**) PaCO_2_ and PtcCO_2_ and (**b**) PaCO_2_ and PetCO_2_ during one-lung ventilation. PetCO_2_, end-tidal carbon dioxide partial pressure; PtcCO_2_, transcutaneous carbon dioxide partial pressure; PaCO_2_, partial pressure of arterial carbon dioxide; SD, standard deviation.

The Bland–Altman analysis during TLV yielded a bias between PaCO_2_ and PtcCO_2_ that was closer to zero than the bias between PaCO_2_ and PetCO_2_ (bias ± SD; −0.7 ± 2.0 mmHg vs. 4.0 ± 5.2 mmHg, *p* < 0.001, Figure 4a,b). The upper and lower LOAs of PtcCO_2_ were narrower than those of PetCO_2_ (−4.6–3.3 mmHg vs. −6.1–14.1 mmHg, Figure 4a,b). During OLV, the bias between PaCO_2_ and PtcCO_2_ was much lower than that between PaCO_2_ and PetCO_2_ (−1.6 ± 6.5 mmHg vs. 14.3 ± 8.4 mmHg, *p* < 0.001, Figure 5a,b). The LOAs of PtcCO_2_ were narrower than those of PetCO_2_ (−14.3–11.2 mmHg vs. −2.2–30.7 mmHg, Figure 5a,b).

## 4. Discussion

This study demonstrates that PtcCO_2_ monitoring allows anesthesiologists to detect increases in PaCO_2_ more accurately than PetCO_2_ monitoring alone, which can prevent excessive hypercapnia and respiratory acidosis. In addition, the study’s results show that PtcCO_2_ is more in agreement with PaCO_2_ compared to PetCO_2_, as indicated by a lower bias and higher precision.

The major concern related to anesthetic techniques for successful non-intubated VATS is to avoid excessive hypercapnia and hypoxemia through effective respiratory management. A previous study reported that an oxygen mask was sufficient to prevent hypoxemia in most non-intubated patients without severe pulmonary comorbidities [17]. In addition, the SpO_2_ levels of patients were generally satisfactory during surgery, and the lowest intraoperative SpO_2_ level was comparable to that of intubated patients [17]. Consistent with this, no patients in the current study experienced persistent hypoxemia during OLV, and the mean PaO_2_ was measured to be 198.3 mmHg. Hypercapnia is a central element of non-intubated thoracic surgery related to hypoventilation due to sedation and OLV [18]. There is a risk of hypercapnic rebreathing effect due to paradoxical respiration that initially occurs and hypoventilation caused by the collapse of the operated lung [5]. Furthermore, while a PaCO_2_ < 70 mmHg is considered “permissive” due to the observed protective effect of improved hemodynamics, ventilation–perfusion match, and reduced inflammatory response, excessive hypercapnia can elevate pulmonary and intracranial pressure and cause cardiac rhythm disturbances [5,19]. Considering that VATS is performed more frequently in older patients susceptible to excessive hypercapnia, the accuracy of CO_2_ monitoring is crucial to maintaining PaCO_2_ within permissive range during non-intubated VATS.

The results of the present study suggest that PtcCO_2_ monitoring is superior to nasal PetCO_2_ monitoring during non-intubated VATS for several reasons. First, as hypo-ventilation occurs during sedation, the partial pressure of CO_2_ in the air exhaled from the lung may represent less than the actual PaCO_2_ concentration, because the sample taken for PetCO_2_ analysis can be diluted with dead space air or supplementary oxygen [20]. Second, spontaneous breathing during the non-intubated VATS might have favorable effects on the functional residual capacity and perfusion of the dependent lung. Nonetheless, the collapsed operated lung and the lateral decubitus position still contribute to increasing the PetCO_2_ to PaCO_2_ gradient [5]. Third, the patient’s abnormal preoperative pulmonary function exacerbates the ventilation–perfusion mismatch [11].

The accuracy of PtcCO_2_ monitoring depends on the patient and technical factors. Two attending anesthesiologists who are proficient at performing PtcCO_2_ monitoring managed all patients. Patient factors such as hypercapnia, low cardiac output, and impaired peripheral perfusion, or the administration of vasoconstrictor agents, may cause artificially low PtcCO_2_ levels [11]. In the current study, 34 of our 43 patients (79.1%) were administered norepinephrine to treat hypotension events. Although the accuracy of PtcCO_2_ monitoring in patients administered vasoconstrictors has been controversial [15,21,22], in our sample, PtcCO_2_ monitoring led to the detection of 49 out of 52 hypercapnia events (PaCO_2_ > 60 mmHg), whereas PetCO_2_ monitoring allowed for the detection of only 10 such events. The expeditious PtcCO_2_-based respiratory management (performed in the 49 of 52 hypercapnia cases) may have contributed to the prevention of the exacerbation of hypercapnia. The result was that persistent severe hypoxemia or hypercapnia with a PaCO_2_ > 80 mmHg requiring conversion to general anesthesia did not occur. Among the 43 patients, 2 were converted to general anesthesia in the current study due to excessive diaphragmatic movement, which might cause unsafe surgery.

Kelly et al. [13] reported that the disagreement between PaCO_2_ and PtcCO_2_ was exacerbated at higher PaCO_2_ levels, suggesting that PtcCO_2_ monitoring is a suboptimal tool. In contrast, the data analyzed in this study show that the mean difference between PaCO_2_ and PtcCO_2_ was as small as −1.6 ± 6.5 mmHg, even when PaCO_2_ increased to approximately 60 mmHg. This discrepancy might be attributable to differences among patient samples. Kelly et al.’s study [13] targeted patients in critical condition with progressive respiratory failure, such as acute pulmonary edema or chronic airway disease. The lung functions of our patients, in contrast, were fairly preserved to maintain spontaneous OLV. The agreement analysis and comparison of hypercapnia detection power strongly indicate that PtcCO_2_ monitoring can help prevent excessive hypercapnia, as it allows for the more accurate detection of PaCO_2_ levels exceeding the threshold of permissive hypercapnia.

While PtcCO_2_ monitoring could offer great advantages, as it continuously surrogates PaCO_2_ and saves efforts for serial ABGA, it still has a limitation of requiring in vivo calibration at first. In vivo calibration has been suggested as a prerequisite step for interpreting PtcCO_2_ with a higher degree of confidence; however, it requires an invasive arterial blood sampling [23]. When arterial cannulation is unnecessary for the surgery, an additional invasive procedure for in vivo calibration might reduce a benefit of a non-invasive CO_2_ measurement technique.

This study had several limitations. First, the inherent limitations of retrospective chart reviews may include unmeasured confounding factors. Second, lung function was preserved in most of our patients, among whom 65.1% had normal pulmonary function test results, and the average predicted forced expiratory volume in 1 s was 94.1%. Hence, the findings of this study cannot not be generalized to patients with severely impaired lung function. Third, although intraoperative administration of dexmedetomidine extended the recovery time from sedation after surgery, missing data during that period prevented us from determining the efficacy of PtcCO_2_ monitoring because patients in the post-anesthesia care unit (PACU) of our hospital generally rely on SpO_2_ monitoring, not on PetCO_2_/PtcCO_2_. Thus, whether a good correlation between PtcCO_2_ and PaCO_2_ is retained during the period of CO_2_ elimination (corresponding to the PACU stay after surgery) should be clarified in future studies.

## 5. Conclusions

We found that PtcCO_2_ monitoring is superior to PetCO_2_ monitoring as a PaCO_2_ surrogate measurement method, because it predicted hypercapnia with higher sensitively and offered a more accurate estimation of PaCO_2._ These findings thus suggest concurrent PtcCO_2_ monitoring to prevent excessive hypercapnia in patients undergoing non-intubated VATS.

## Figures and Tables

**Table 1:** Patient demographic and procedure characteristics (n = 43).

Variables	
Height (cm)	160.5 ± 9.3
Weight (kg)	59.4 ± 8.6
BMI (kg/m^2^)	23.0 ± 2.1
Age (years)	63.7 ± 10.7
Sex (male: female), n	20:23
ASA physical status (I: II: III), n	1:31:11
Pulmonary function test, n (normal: restrictive: obstructive: mixed type)	28:2:8:5
FVC, % predicted	93.6 ± 12.7
FEV1, % predicted	94.0 ± 12.8
FEV1/FVC (%)	75.4 ± 7.1
Patients who needed norepinephrine, n (%)	34 (79.1%)
Surgery time, min	156.9 ± 57.0
Anesthetic time, min	194.9 ± 53.3
Types of thoracic procedure, n (%)	
Left upper lobectomy	8 (18.6%)
Left lower lobectomy	5 (11.6%)
Right upper lobectomy	21 (48.8%)
Right lower lobectomy	8 (18.6%)
Bilobectomy	1 (2.3%)

Values are presented as mean ± standard deviation, number, or number (percentage). BMI, body mass index; ASA, American Society of Anesthesiologists; FEV1, forced expiratory volume in 1 s; FVC, forced vital capacity.

**Table 2:** Physiological data measured during TLV and OLV.

	TLV	OLV	*p*-Value *
pH	7.3 ± 0.0	7.2 ± 0.1	< 0.001
PaO_2_ (mmHg)	261.4 ± 138.1	198.3 ± 118.0	0.011
SpO_2_ (%)	98.8 ± 2.0	97.4 ± 2.9	0.003
PaCO_2_ (mmHg)	46.1 ± 7.3	59.4 ± 10.2	<0.001
PetCO_2_ (mmHg)	42.1 ± 6.5	45.1 ± 7.9	0.030
PtcCO_2_ (mmHg)	46.8 ± 7.5	61.0 ± 10.5	<0.001

Values are presented as mean ± standard deviation. * A *t*-test was used to compare variables measured during TLV and OLV. PaO_2_, partial pressure of oxygen; SpO_2_, saturation of percutaneous oxygen; PaCO_2_, partial pressure of arterial carbon dioxide; PetCO_2_, end-tidal carbon dioxide partial pressure; PtcCO_2_, transcutaneous carbon dioxide partial pressure; TLV, two-lung ventilation; OLV, one-lung ventilation.

## Data Availability

The datasets are available upon reasonable request to the corresponding author.

## References

[1] Wu C.Y., Chen J.S., Lin Y.S., Tsai T.M., Hung M.H., Chan K.C., Cheng Y.J. (2013). Feasibility and safety of nonintubated thoracoscopic lobectomy for geriatric lung cancer patients. Ann. Thorac. Surg..

[2] Pompeo E., Mineo D., Rogliani P., Sabato A.F., Mineo T.C. (2004). Feasibility and results of awake thoracoscopic resection of solitary pulmonary nodules. Ann. Thorac. Surg..

[3] Pompeo E., Rogliani P., Tacconi F., Dauri M., Saltini C., Novelli G., Mineo T.C., Awake Thoracic Surgery Research Group (2012). Randomized comparison of awake nonresectional versus nonawake resectional lung volume reduction surgery. J. Thorac. Cardiovasc. Surg..

[4] Pompeo E., Tacconi F., Mineo D., Mineo T.C. (2007). The role of awake video-assisted thoracoscopic surgery in spontaneous pneumothorax. J. Thorac. Cardiovasc. Surg..

[5] Gonzalez-Rivas D., Bonome C., Fieira E., Aymerich H., Fernandez R., Delgado M., Mendez L., de la Torre M. (2016). Non-intubated video-assisted thoracoscopic lung resections: The future of thoracic surgery?. Eur. J. Cardiothorac. Surg..

[6] He J., Liang H., Wang W., Akopov A., Aiolfi A., Ang K.L., Bertolaccini L., Cai K., Cao Q., Chen B. (2021). Tubeless video-assisted thoracic surgery for pulmonary ground-glass nodules: Expert consensus and protocol (Guangzhou). Transl. Lung Cancer Res..

[7] Kiss G. (2020). Technical issues and patient safety in nonintubated thoracic anesthesia. Thorac. Surg. Clin..

[8] Carmi U., Kramer M.R., Zemtzov D., Rosengarten D., Fruchter O. (2011). Propofol safety in bronchoscopy: Prospective randomized trial using transcutaneous carbon dioxide tension monitoring. Respiration.

[9] Heuss L.T., Chhajed P.N., Schnieper P., Hirt T., Beglinger C. (2004). Combined pulse oximetry/cutaneous carbon dioxide tension monitoring during colonoscopies: Pilot study with a smart ear clip. Digestion.

[10] Monaco F., Nickerson B.G., McQuitty J.C. (1982). Continuous transcutaneous oxygen and carbon dioxide monitoring in the pediatric ICU. Crit. Care Med..

[11] Oshibuchi M., Cho S., Hara T., Tomiyasu S., Makita T., Sumikawa K. (2003). A comparative evaluation of transcutaneous and end-tidal measurements of CO_2_ in thoracic anesthesia. Anesth. Analg..

[12] Li H., Huang D., Qiao K., Wang Z., Xu S. (2019). Feasibility of non-intubated anesthesia and regional block for thoracoscopic surgery under spontaneous respiration: A prospective cohort study. Braz. J. Med. Biol. Res..

[13] Kelly A.M., Klim S. (2011). Agreement between arterial and transcutaneous PCO_2_ in patients undergoing non-invasive ventilation. Respir. Med..

[14] Nicolini A., Ferrari M.B. (2011). Evaluation of a transcutaneous carbon dioxide monitor in patients with acute respiratory failure. Ann. Thorac. Med..

[15] De Oliveira G.S., Ahmad S., Fitzgerald P.C., McCarthy R.J. (2010). Detection of hypoventilation during deep sedation in patients undergoing ambulatory gynaecological hysteroscopy: A comparison between transcutaneous and nasal end-tidal carbon dioxide measurements. Br. J. Anaesth..

[16] Akoglu H. (2018). User’s guide to correlation coefficients. Turk. J. Emerg. Med..

[17] Sunaga H., Blasberg J.D., Heerdt P.M. (2017). Anesthesia for nonintubated video-assisted thoracic surgery. Curr. Opin. Anaesthesiol..

[18] Janík M., Juhos P., Lučenič M., Tarabová K. (2021). Non-intubated thoracoscopic surgery-pros and cons. Front. Surg..

[19] Contreras M., Masterson C., Laffey J.G. (2015). Permissive hypercapnia: What to remember. Curr. Opin. Anaesthesiol..

[20] Nelson D.B., Freeman M.L., Silvis S.E., Cass O.W., Yakshe P.N., Vennes J., Stahnke L.L., Herman M., Hodges J. (2000). A randomized, controlled trial of transcutaneous carbon dioxide monitoring during ERCP. Gastrointest. Endosc..

[21] Rodriguez P., Lellouche F., Aboab J., Buisson C.B., Brochard L. (2006). Transcutaneous arterial carbon dioxide pressure monitoring in critically ill adult patients. Intensive Care Med..

[22] Zhang H., Wang D.X. (2015). Noninvasive measurement of carbon dioxide during one-Lung ventilation with low tidal volume for two hours: End-tidal versus transcutaneous techniques. PLoS ONE.

[23] Cuvelier A., Grigoriu B., Molano L.C., Muir J.F. (2005). Limitations of transcutaneous carbon dioxide measurements for assessing long-term mechanical ventilation. Chest.

